# Viral protein R (Vpr)-induced neuroinflammation and its potential contribution to neuronal dysfunction: a scoping review

**DOI:** 10.1186/s12879-023-08495-3

**Published:** 2023-08-06

**Authors:** Monray Edward Williams, Aurelia A. Williams, Petrus J.W. Naudé

**Affiliations:** 1 Human Metabolomics, North-West University, Potchefstroom, South Africa; 2 Department of Psychiatry and Mental Health, University of Cape Town, Cape Town, South Africa; 3 Neuroscience Institute, University of Cape Town, Cape Town, South Africa

**Keywords:** HIV-associated neurocognitive disorders, Vpr, Neuroinflammation, Neuronal dysfunction and neurotoxicity

## Abstract

HIV-associated neurocognitive disorders (HAND) are the result of the activity of HIV-1 within the central nervous system (CNS). While the introduction of antiretroviral therapy (ART) has significantly reduced the occurrence of severe cases of HAND, milder cases still persist. The persistence of HAND in the modern ART era has been linked to a chronic dysregulated inflammatory profile. There is increasing evidence suggesting a potential role of Viral protein R (Vpr) in dysregulating the neuroinflammatory processes in people living with HIV (PLHIV), which may contribute to the development of HAND. Since the role of Vpr in neuroinflammatory mechanisms has not been clearly defined, we conducted a scoping review of fundamental research studies on this topic. The review aimed to assess the size and scope of available research literature on this topic and provide commentary on whether Vpr contributes to neuroinflammation, as highlighted in fundamental studies. Based on the specified selection criteria, 10 studies (6 of which were cell culture-based and 4 that included both animal and cell culture experiments) were eligible for inclusion. The main findings were that (1) Vpr can increase neuroinflammatory markers, with studies consistently reporting higher levels of TNF-α and IL-8, (2) Vpr induces (neuro)inflammation via specific pathways, including the PI3K/AKT, p38-MAPk, JNK-SAPK and Sur1-Trpm4 channels in astrocytes and the p38 and JNK-SAPK in myeloid cells, and (3) Vpr-specific protein amino acid signatures (73R, 77R and 80A) may play an important role in exacerbating neuroinflammation and the neuropathophysiology of HAND. Therefore, Vpr should be investigated for its potential contribution to neuroinflammation in the development of HAND.

**Supplementary Information:**

The online version contains supplementary material available at 10.1186/s12879-023-08495-3.

## Introduction

HIV-1 causes a spectrum of neurocognitive deficits known as HIV-associated neurocognitive disorders (HAND) [1]. According to the Trojan horse hypothesis, HIV-1 can breach the blood-brain barrier by infiltrating monocytes that later transform into macrophages, allowing the virus to enter the central nervous system (CNS) [2]. Once inside the CNS, HIV-1 utilises various mechanisms to induce neuronal damage, thereby contributing to the development of HAND [2]. Several viral proteins are involved in HIV-1 associated neuropathogenesis, including; glycoprotein 120 (gp120) [3–5], transactivator of transcription (Tat) [6–8], negative factor (Nef) [9, 10], regulator of expression of virion proteins (Rev) [11] and viral protein R (Vpr) [12, 13].

Increasing evidence suggests that Vpr serves as an important multifunctional accessory protein of HIV-1 [14, 15]. Vpr is a fourteen-kilodalton basic protein comprised of 96 amino acids and consists of three amphiphilic helices [16]. These bundled α-helices extend to the amino acid regions 17–33, 38–50, and 55–77 and are flanked by unstructured flexible N-and C-terminal domains that are negatively or positively charged, respectively [16]. Vpr plays a critical role in HIV-1 pathogenesis [17], including infection of dividing as well as non-dividing cells (myeloid and resting T-cells) through various effects (nuclear localization, cell cycle arrest, apoptosis, and other effects due to DDB1–Cul4-associated factor 1 (DCAF)-1 binding), as well as transactivation of host and viral genes [15, 18]. Moreover, evidence suggests Vpr’s involvement in HIV-1 neuropathogenesis [19]. Vpr is a transducing protein capable of being internalised by glial cells and neurons [20, 21], and can negatively affect neuronal health. With the harbouring of HIV-1 within the CNS, extracellular Vpr damages neurons by elevating caspase 3, caspase 6 and cytochrome C levels, ultimately leading to neuronal apoptosis [22]. Extracellular Vpr may also adversely affect the blood-brain barrier [23], potentially increasing the influx of infected cells into the CNS. In addition, microglia and astrocytes that are exposed to Vpr in vitro exhibit changes in metabolism, reactive oxygen species, lactate production, adenosine triphosphate, glutathione and inflammation [24, 25], all of which are recognized mechanisms contributing to HIV-1 neuropathogenesis [19, 20, 22, 26]. Specific Vpr amino acids have been linked to the clinical presentation of HAND in people living with HIV (PLHIV) [12].

The role of Vpr in the development of HAND is of interest because, similar to Tat [27], even in the absence of active viral replication and with the effective use of antiretroviral therapy (ART), Vpr can still be detected in the periphery and CNS of PLHIV [22, 28, 29]. Therefore, Vpr may have a functional role in the development of HAND in the modern ART era.

Although the more clinically severe forms of HAND have diminished with the introduction of ART, milder forms continue to persist [30]. It is hypothesised that the persistence of HAND in the ART era is due to ongoing immune activation and low-grade inflammation experienced by PLHIV [31]. The fact that extracellular Vpr can be detected in peripheral blood regardless of viral suppression [29] and is present in the CNS [32], suggests that Vpr might contribute to the dysregulated inflammatory profile observed in PLHIV in the modern ART era. However, the potential relationship between Vpr and neuroinflammation has yet to be clearly defined. Therefore, the primary aim of this scoping review was to determine the extent of the available evidence by reviewing all literature on this topic to date and to determine the value of undertaking a full systematic review and meta-analysis to provide commentary on whether Vpr contributes to neuroinflammation as highlighted in fundamental studies. The secondary aims of this study were to explore the impact of Vpr presence on inflammatory markers and pathways, as well as to assess whether variations in Vpr amino acids influence neuroinflammation and neuronal health.

## Methods

### Study design

This scoping review aimed at synthesising the extant literature of basic/fundamental studies investigating Vpr-induced neuroinflammation to provide commentary into its potential contribution to neuronal damage. However, the available evidence on this topic remains limited, making it uncertain whether a comprehensive systematic review and meta-analysis can be performed. Therefore, conducting a scoping review is a crucial initial step to determine the feasibility and value of undertaking a full systematic review and meta-analysis. This scoping review has been conducted in accordance with the Preferred Reporting Items for Systematic Reviews and Meta-Analyses for scoping reviews (PRISMA-ScR) guidelines.

### Eligibility criteria

Studies were included if they measured Vpr-induced inflammatory markers from brain cells. Therefore, these included investigations of Vpr-exposed and/or transfected neuronal cells, brain microvascular endothelial cells, astrocytes, microglia, and animal brain tissues. Studies that measured Vpr-induced inflammation from monocytes/macrophages had to directly associate the expression levels with neuronal damage to be included. To ensure comparability, marker measurements needed to be conducted using enzyme-linked immunosorbent assay (ELISA) or similar cytokine arrays (e.g., Bioplex, proteome profiler array), transcript-specific polymerase chain reaction (PCR), immunocytochemistry, immunohistochemistry, and immunofluorescence. Studies were excluded if they investigated inflammation from any other Vpr-exposed/transfected cells that were unrelated to the CNS. Purely clinical studies were excluded. Although efforts were made to exclude all clinical studies that investigated the relationship between Vpr and HAND and/or Vpr and peripheral inflammation, certain studies had both fundamental (e.g., cell culture models) and clinical (e.g., human brain tissue or CSF) designs. However, for the purpose of this review, only the fundamental and preclinical data (in vivo and in vitro) were extracted, and all clinical data from these studies were excluded. Clinical studies investigating Vpr focused solely on the correlation between specific Vpr amino acids and neurocognitive impairment [12, 33], or the relationship between Vpr presence in blood and elevated levels of specific immune markers; Soluble intercellular adhesion molecule (sICAM)-1 and C-C motif ligand (CCL)2 [29]. To date, no clinical study has explored the relationship between Vpr, neuroinflammation and neurocognitive impairment. Therefore, clinical studies were not included in this review.

### Data sources

A comprehensive search was conducted in the PubMed, Scopus, and Web of Science databases to identify relevant studies. The search included all studies published until 27/02/2023, with no restrictions on publication dates. Only studies published in English were included. The detailed search strategy for each database can be found in the supplementary file. The following search terms were applied to PubMed: Viral protein R [tw] OR Gene Products, vpr [mh]) AND (HIV associated neurocognitive disorders [mh] OR HAND [tw] OR neurocognitive [tw] OR cogniti* [tw] OR Neuropsychological Tests [mh] OR neuronal damage [tw] OR neuronal apoptosis [tw] OR inflammation [mh] OR Cytokines [mh] OR Chemokines [mh] OR Neurogenic Inflammation [mh] OR neuroinflammation [tw] OR TNF [tw] OR Interleukins [mh] OR interleukins [tw] OR Microglia [mh] OR Monocytes [mh] OR Microglia [mh] OR microglia [tw] OR Monocytes [mh] OR monocyte* [tw] OR sCD163 [tw] OR sCD14 [tw] OR sCD40 [tw] OR Neopterin [mh] OR Interferons [mh].

**Fig. 1:**
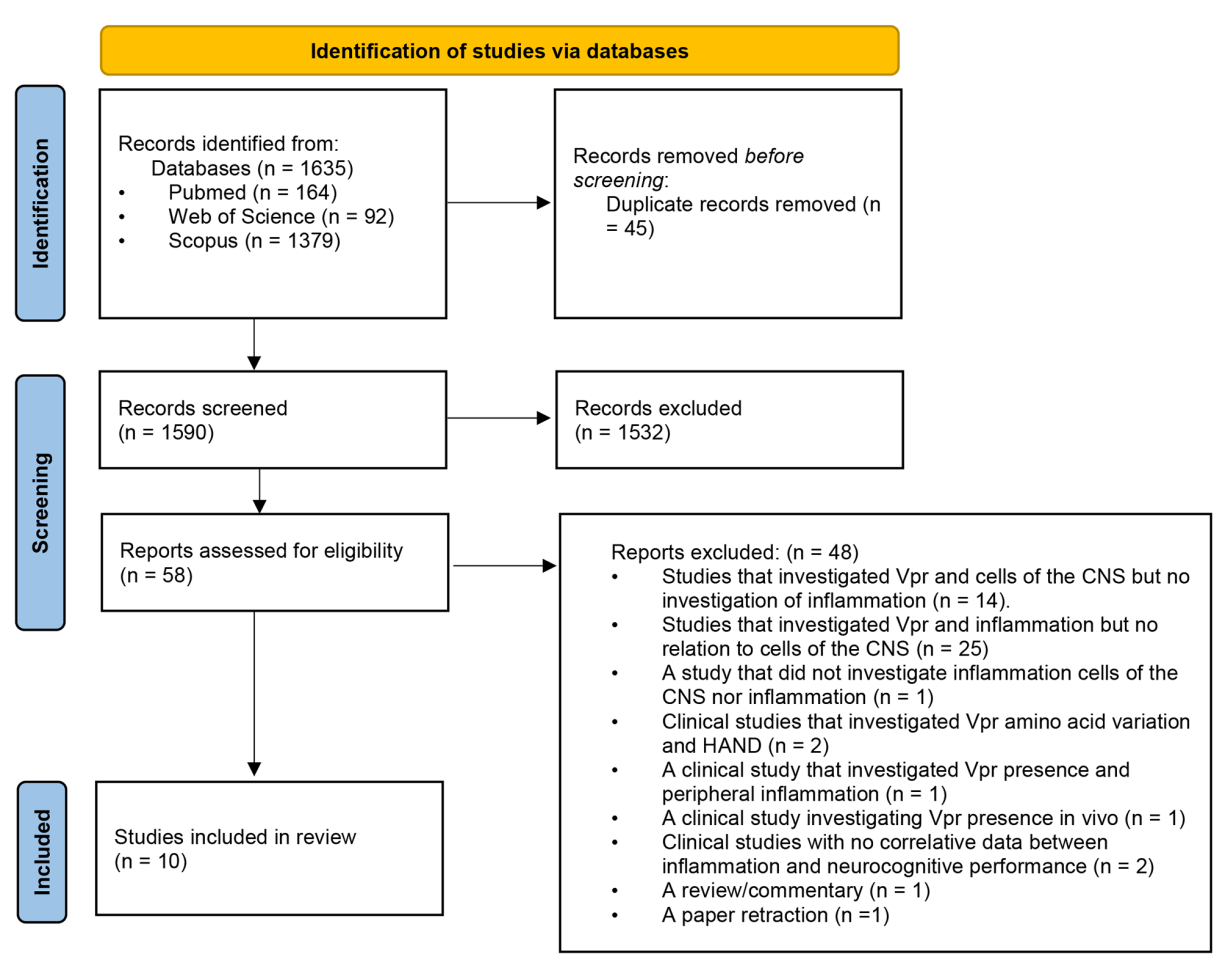
Preferred Reporting Items for Systematic Reviews and Meta-Analyses (PRISMA) flow diagram

Furthermore, reference sections were manually searched, and, where required, the authors of the included studies were contacted for any relevant studies on the topic. The search strategy and the retrieved articles are shown in Fig. 1.

### Data selection

All articles were retrieved and loaded onto a single database using a reference manager (EndNote X9, Clarivate, PA, USA). Two authors, MEW and PJWN independently identified studies meeting the inclusion criteria. Discrepancies were resolved through consensus between the two authors. If a resolution could not be reached, a third author or senior researcher involved in the study was consulted for arbitration. Cohen’s Kappa was used to measure inter-rater agreement. Data extraction encompassed the type of investigation model, investigated markers, assays, and key findings. The extracted data were subsequently categorized and classified based on relevant variables (Tables 1 and 2).

**Table 1:** Summary and key findings from studies investigating Vpr-induced neuroinflammation from myeloid and microvascular endothelial cells

Vpr status	Model	Markers Investigated	Assay	Key findings for marker levels	Other key findings	References
Viral protein R (Vpr)+	Microglia	Chemokine (C-C motif) ligand CCL5/RANTES, Interferon gamma-induced protein 10 (IP-10) and CCL2	Enzyme-linked immunosorbent assay (ELISA)	↑CCL5	1. Vpr was required for efficient viral replication and chemokine expression in microglia.	[37]
Vpr+	Monocyte-derived macrophages (MDMs)	Interleukin (IL)-1β, IL-8 and Tumour necrosis factor (TNF)-α	ELISA	↑IL-1β, IL-8 and TNF-α	1. Vpr presence activated the p38 and stress-activated protein kinase (SAPK)/c-Jun N-terminal kinase (JNK) more than HIV-1 (Vpr deleted mutant)-infected MDMs. 2. The increased expression of IL-1β, IL-8 and TNF-α increased the neurotoxicity of primary neurons	[36]
Vpr+	Macrophages/microglia	IL-1β	Immunofluorescence	↑IL-1β	1. Pseudotyped viruses containing Vpr led to a significant reduction in cell viability in differentiated THP-1 cells and microglia. 2. Increased NOD-, LRR- and pyrin domain-containing protein 3 (NLRP3), caspase-1, and IL-1β expression was evident in HIV-1 Vpr transgenic mice compared to wild-type littermates, following systemic immune stimulation 3. Treatment with the caspase-1 inhibitor, VX-765, suppressed NLRP3 expression with reduced IL-1β expression and associated neuroinflammation which results in an improvement in neurobehavioral deficits in Vpr transgenic animals.	[32]
Vpr+	Myeloid	TNF-α and IFN-α	Polymerase chain reaction (PCR)	↑IFN-α and TNF-α	1. Vpr was found in human brain tissue and peripheral blood mononuclear cells (PBMCs) 2. Vpr transcripts were found in the human brain and were significantly higher among HIV-associated dementia (HAD) patients (59%) compared with non-demented (ND) patients (31%). 3. Vpr amino acid signature 77Q was largely present in HAD and 77R was present in ND participants 4. Myeloid transfected cells with Vpr 77R induced TNF-α and IFN-α significantly more than Vpr 77Q and Vpr (-). 5. Supernatants derived from Vpr + transfected cells and added to human fetal neurons led to significant reductions in neuronal viability compared with supernatants from the Vpr(-) transfected cells.	[39]
					6. Interestingly, Vpr R77 showed the greatest neurotoxicity, in line with the neuroimmune response. 7. Human fetal microglia were exposed to Vpr peptides revealing that Vpr77R peptide activated greater IFN-α compared to Vpr 77Q or mock exposed glia cell. 8. A reduced number of neurons were found in animals implanted with the full-length Vpr and Vpr 77R. 9. Both full-length Vpr and Vpr77R caused significantly increased rotary behaviour compared with Phosphate-buffered saline (PBS)-implanted animals 10. Findings show that in that Vpr containing 77R was more neurovirulent compared with the 77Q peptide or controls.	
Vpr+	Microvascular endothelial cells (MVECs)	TNF-α	ELISA	↑TNF-α	1. Vpr induced increased apoptosis in MVECs	[23]
Vpr+	Monocytes	IL-6	PCR	↓IL-6	1. Lower IL-6 transcript levels were found in the basal ganglia (BG), cortex, and hindbrain of Vpr Transgenic animals compared with wild-type controls	[22]

The table is presented according to the findings presented in Sect. 3.3

Abbreviations: Basal ganglia (BG), Chemokine (C-C motif) ligand, Enzyme-linked immunosorbent assay (ELISA), HIV-associated dementia (HAD), Interferon gamma-induced protein 10 (IP-10), Interleukin (IL), Microvascular endothelial cells (MVECs), Monocyte-derived macrophages (MDMs), NOD-, LRR- and pyrin domain-containing protein 3 (NLRP3), Non-demented (ND), Peripheral blood mononuclear cells (PBMCs), Phosphate-buffered saline (PBS), Polymerase chain reaction (PCR), Stress-activated protein kinase (SAPK)/c-Jun N-terminal kinase (JNK), Tumour necrosis factor (TNF)-α, Viral protein R (Vpr)+

**Table 2:** Summary and key findings from studies investigating Vpr-induced neuroinflammation from astrocytes

Vpr status	Model	Markers Investigated	Assay	Key findings for marker levels	Other key findings	Ref
Viral protein R (Vpr)+	Human fetal astrocytes (HFAs)	Interleukin (IL)-6, IL-8, C–C motif chemokine ligand (CCL)-2 and migration inhibitor factor (MIF)	Cytokine Array	↑ IL-6, IL-8, C–CCL-2 and MIF	1. SK-N-SH neuroblastoma cells exposed to recombinant (r)Vpr or mutant rVpr (73R and 80 A) conditioned medium decreased synthesis of Glutathione and increased apoptosis	[26]
Vpr+	HFAs	CCL5	Polymerase chain reaction (PCR) and Immunocytochemistry	↑CCL5	1. Increased CCL5 induction mediated by transcription factors nuclear factor kappa B (NF-κB) and Activator protein (AP)-1 and involved the p38- Mitogen-activated protein kinase (MAPK) and Phosphoinositide 3-kinase (PI3K)/Akt pathway.	[25]
Vpr+	HFAs	IL-6 and IL-8	Polymerase chain reaction (PCR)	↑IL-6 and IL-8	1. Increased levels of IL-6 and IL-8 were by the increased activation of transcription factors NF-κB, AP-1 and C/EBP-δ via upstream protein kinases PI3K/Akt, p38-MAPK and Jun N-terminal kinases (Jnk)-MAPK	[38]
Vpr+	Astrocytes	Toll-like receptor (TLR)4 and Tumour necrosis factor (TNF)-α,	Immunohistochemistry	↑ TLR4 and TNF-α	1. Vpr was colocalized with upregulated expression of TLR4, TNF-α, NF-κB, and sulfonylurea receptor 1 - Transient receptor potential (Trp) melastatin 4 (Sur1-Trpm4) in astrocytes of brain tissue from HIV-infected patients as well as in mice brain tissue. 2. Increased level of TLR4, TNF-α, NF-κB was by increased expression of Sur1-Trpm4 channel in astrocytes	[40]
Vpr+	Astrocytes	IL-6 and IL-1β	PCR	↓ IL-6 and IL-1β	1. Soluble Vpr was neurotoxic to primary human and rat fetal neurons in a concentration-dependent manner, as indicated by loss of β-tubulin expression 2. Findings for IL-1β levels from astrocytes were variable compared to monocytic cells 3. IL-6 were lower in both astrocytes and monocytic cells	[22]

The table is presented according to the findings presented in Sect. 3.3

Abbreviations: Activator protein (AP)-1, C–C motif chemokine ligand (CCL), Human fetal astrocytes (HFAs), Interleukin (IL), Migration inhibitor factor (MIF), Mitogen-activated protein kinase (MAPK), Nuclear factor kappa B (NF-κB), Phosphoinositide 3-kinase (PI3K), Polymerase chain reaction (PCR), Sulfonylurea receptor 1 -Transient receptor potential (Trp) melastatin 4 (SUR1-TRPM4), Toll-like receptor (TLR)4, Tumour necrosis factor (TNF)-α and Viral protein R (Vpr)+

### Quality assessment

The quality of the included studies was assessed by MEW. The quality criterion was adopted from the Joanna Briggs Institute (JBI) critical appraisal tools. Here we have amended the JBI critical appraisal tools by implementing a Likert scale [34] to provide a quantitative measure of study quality. We adopted and amended the quality questions relevant to in vitro and/or in vivo studies. These included 4 questions related to study design; (1) Is it clear in the study what is the ‘cause’ and what is the ‘effect’ (i.e., there is no confusion about which variable comes first)? (2) was there a control group? (3) were there multiple measurements of the outcome both pre and post intervention/exposure? and (4) were outcomes measured in a reliable way? This refers to whether the studies ensured the accuracy, consistency, and reproducibility of measurements by conducting independent replication studies and employing other appropriate measures. Each question was rated for 0 = no, 1 = partly and 2 = yes. All studies with summative ratings between 6 and 8 were classified as high quality. Studies with ratings between 3 and 5 were considered intermediate quality, and between 0 and 2 as low quality (Supplementary Table 1).

## Results

### Study characteristics

The search strategy yielded a total of 1635 research studies, as indicated in Fig. 1. Duplicates (n = 45) were removed, resulting in 1590 studies. Thereafter, abstracts and titles were screened and a total of 1532 studies were excluded. Of the remaining 58 studies, full-text articles were assessed, and an additional 48 were excluded as described in Fig. 1. The kappa was 0.95 which indicates almost perfect agreement [35]. Using the specified selection criteria, 10 fundamental studies were eligible for inclusion.

From the included studies, various sample types were investigated, including MVECs, HFAs, HAs, HFNs, mice and rat brain tissue, MFN, myeloid cells (human monocytes, MDMs, and primary human microglia), neuroblastoma cells, and primary neurons. Additionally, immune marker measurements were conducted using a range of tools, such as ELISA [23, 36, 37], cytokine arrays [26, 38], immune marker PCR [22, 25, 32, 36, 38, 39], immunocytochemistry [25, 38], immunohistochemistry [40] and immunofluorescence [32].

### Quality assessment of the included studies

All studies were considered high quality, with clear descriptions of cause and expected outcomes, a control group, the use of multiple investigations (e.g., inflammation and pathway activation) and appropriate measurements and techniques to answer the research question (Supplementary Table 1).

### Key findings

In this study, we performed a scoping review of fundamental research focused on Vpr-induced neuroinflammation. By applying specific selection criteria, we identified 10 eligible studies for review, including 6 studies conducted in cell culture settings and 4 studies involving both animal and cell culture experiments. These investigations primarily focused on assessing neuroinflammation levels in cells predominantly found in the CNS, utilizing various methodologies. The review revealed several noteworthy findings that warrant attention.

**Fig. 2:**
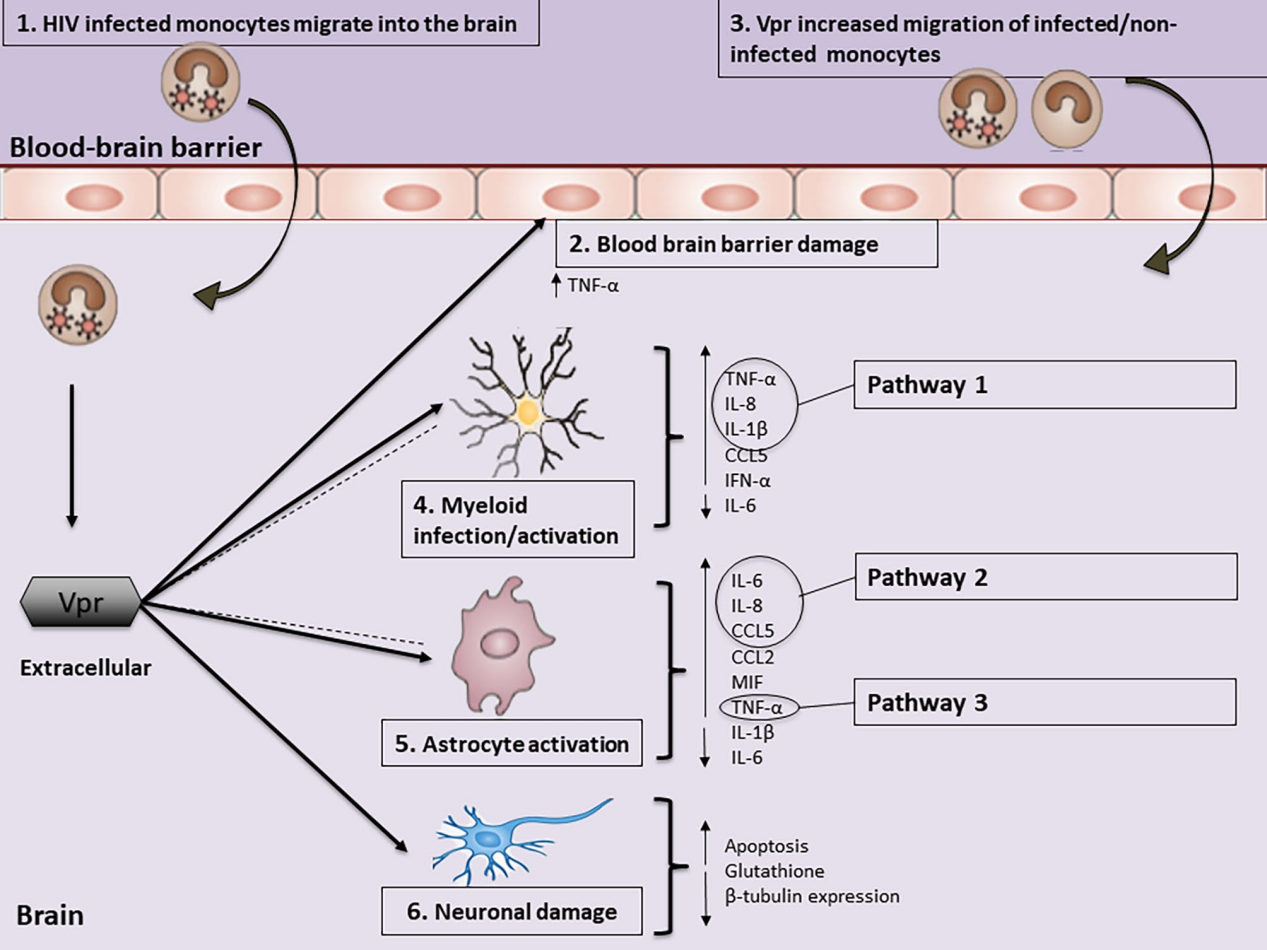
Vpr-induced neuroinflammation. **(1)** Vpr is introduced into the central nervous system (CNS) through HIV-1 infected cells including monocytes. The secreted extracellular Vpr that is present in the CNS stimulates intracellular pro-inflammatory pathways in myeloid, astrocytes and brain microvascular endothelial cells (BMVECs). **(2)** Extracellular Vpr causes damage to the blood-brain barrier (BBB) and increases the release of TNF-α from BMVECs **(3)** which results in increased migration of cells into the CNS. **(4)** Vpr increases the release of the inflammatory markers TNF-α, IL-8, IL-1β, CCL5, and IFN-α and lowers IL-6 from activated myeloid cells. **(5)** Vpr increases the release of inflammatory markers IL-6, IL-8, CCL5, CCL2, MIF and TNF-α and lowers IL-1β and IL-6 levels from activated astrocytes. In addition, myeloid and astrocytes also release extracellular Vpr into the cerebrospinal fluid (CSF) (dotted lines). **(6)** Vpr also directly induces neuronal damage. Vpr-associated neuroinflammation is induced by myeloid cells (monocyte-derived macrophages, MDMs) and astrocytes via pathway 1 (Fig. 3A) and astrocytes via pathway 2 (Fig. 3B)

First, based on the reviewed fundamental studies, it is evident that Vpr exposure/transfection contributes to the level of neuroinflammation. These studies investigated inflammation levels when myeloid and microvascular endothelial cells were transfected/exposed to Vpr (Table 1). Si and colleagues (2002) reported that Vpr-positive infected microglia cells exhibited significantly higher C-C chemokine ligand (CCL)5 compared to Vpr-negative and mock-infected microglia [37]. Further, another study found that HIV-1-infected (Vpr-positive wild-type) MDMs resulted in higher production of interleukin (IL)-1β, IL-8 and tumour necrosis factor (TNF)-α at the transcriptional and/or protein levels as compared to HIV-1-infected MDMs (Vpr-deleted mutant) [36]. Supporting the findings for IL-1β, Vpr-exposed human macrophages demonstrated a substantial increase in IL-1β protein expression [32]. A similar trend was noted with regard to other types of inflammatory markers as shown in previous work by Na and colleagues (2011), who reported that myeloid Vpr-positive transfected cells produced significantly more Interferon (IFN)-α and TNF-α compared to Vpr-negative mock-transfected cells [39]. In a study that investigated Vpr-treated MVECs, TNF-α secretion was found to be elevated compared to controls [23]. Additionally, another study indicated that IL-6 transcript levels were lower in Vpr-stimulated monocytes compared to unstimulated cells, and IL-6 levels decreased in a concentration-dependent manner with increased Vpr [22] (Table 1; Fig. 2).

The reviewed studies also investigated the levels of inflammation when astrocytes were exposed/transfected with Vpr. Significant differences in the levels of inflammatory markers were reported when compared to control cells [22, 26, 38, 40] (Table 2; Fig. 2). Ferrucci and colleagues (2013) reported that when HFAs were treated with recombinant Vpr (rVpr), higher levels of IL-6, IL-8, CCL2, and migration inhibitor factor (MIF) were induced as compared to untreated HFAs [26]. Gangwani and colleagues (2013) reported that Vpr-transfected astrocytes secreted increased CCL5 at the genetic and protein levels compared to mock-transfected controls [25]. Gangwani and Kumar (2015) further investigated additional markers and reported that Vpr-transfected astrocytes secreted increased IL-6 and IL-8 protein levels at all-time points assayed (6, 12, 24, 48 and 72 h) with a peak at 72 h as compared to mock-transfected controls [38] (Table 2; Fig. 2).

In a study with astrocytes from brain tissue of HIV-infected patients, higher Vpr expression was colocalized with upregulated expression of TLR4, TNF-α, nuclear factor Kappa B (NF-κB), and sulfonylurea receptor 1 (Sur1)- transient receptor potential melastatin 4 (Trpm4) [40] (Table 2; Fig. 2). NF-κB is a transcription factor [41, 42], TLR4 is a pattern recognition receptor [43] and Sur1-Trpm4 is an ion channel[44], all of which are known to be involved in pathways of HIV-1 induced inflammation. A study by Jones and colleagues (2007) found that in HIV-1-infected Vpr-stimulated astrocytes, IL-1β transcript levels were lower compared to unstimulated controls, and IL-1β levels decreased in a concentration-dependent manner with increased Vpr [22]. The same study found that in HIV-1-infected Vpr-stimulated astrocytes, IL-6 levels were lower at Vpr concentrations of 1nM and 10nM, respectively, and IL-6 was only higher at a Vpr concentration of 100 nM compared to control cells [22] (Table 2). Collectively, these findings support the premise that Vpr is important in mediating neuroinflammation.

Secondly, the reviewed studies investigated various inflammatory markers, including CCL2, CCL5, IL-1β, IL-6, IL-8, IFN-α, IP-10, MIF, NF-κB, TLR4 and TNF-α. Among these markers, the majority of the studies focused on TNF-α (n = 4), IL-6 (n = 3), IL-8 (n = 3) and IL-1β (n = 3). Based on the most commonly investigated markers (see summary Tables 1 and 2), we considered markers to be noteworthy when more than two independent studies consistently reported a similar trend in the levels of the inflammatory marker in the presence of Vpr. The studies consistently reported increased TNF-α levels in cell cultures with MVECs [23], myeloid cells [39], MDMs [36] and astrocytes [40] that were exposed to Vpr as compared to mock-treated/transfected cells (Fig. 2). Similarly, IL-8 levels from MDMs [36] and HFAs [26, 38] cell cultures that were exposed to Vpr were consistently higher, compared to mock-treated/transfected cells. However, the findings for IL-1β and IL-6 were inconsistent, despite more than two independent studies reporting on them (Tables 1 and 2). Specifically, IL-1β levels in astrocytes and MDMs were found to be lower when treated/transfected with Vpr [22, 36], while microglia exhibited higher levels [32] (Tables 1 and 2). Similarly, the findings regarding IL-6 in experiments with cell cultures were contradictory, with lower levels observed in in astrocytes and monocytes [22], and higher levels observed in HFAs [26, 38] when treated/transfected with Vpr (Fig. 2). Therefore, the higher levels of TNF-α and IL-8 may be noteworthy markers induced by Vpr for future investigations (Tables 1 and 2).

**Fig. 3:**
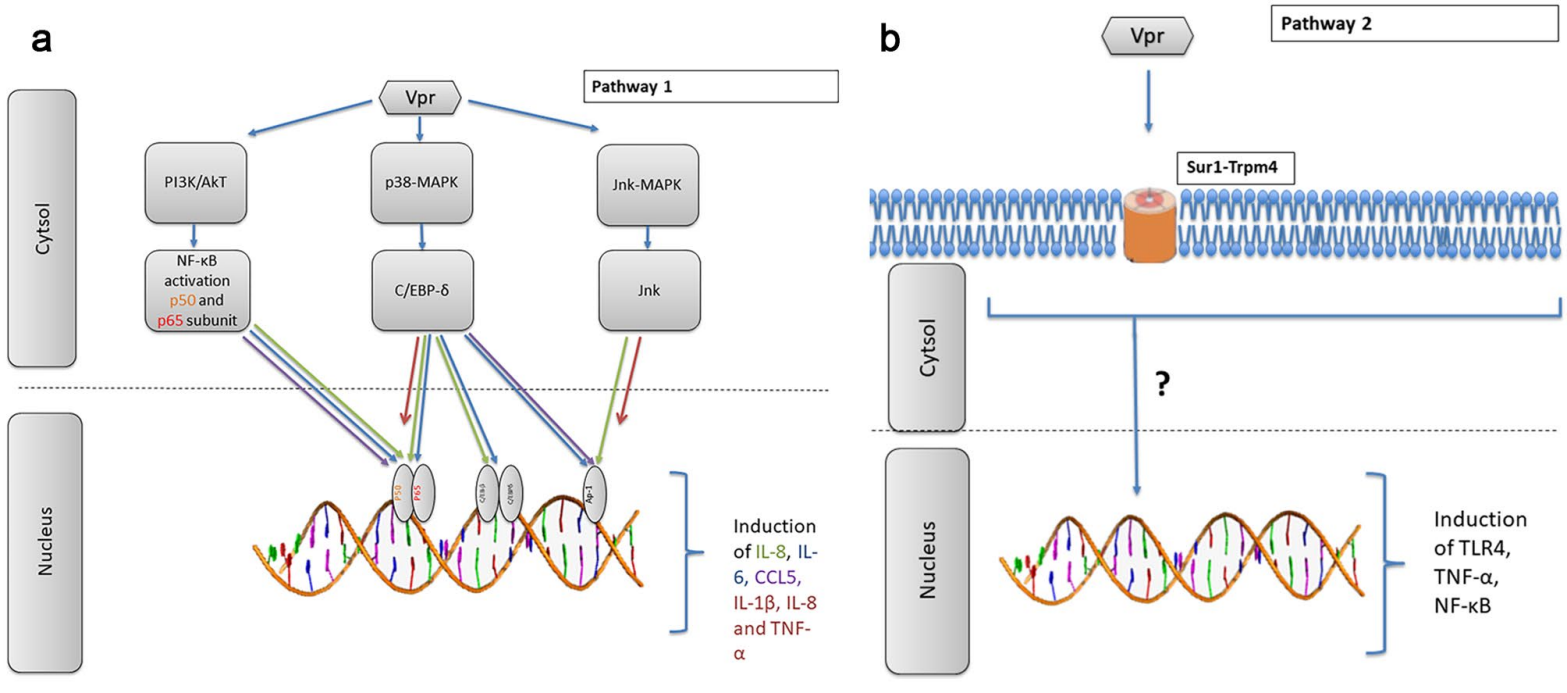
**A:** Pathway 1 of Vpr-induced neuroinflammation. Pathway 1 indicates the Vpr increased induction of IL-6, IL-8 and CCL5 via the PI3K/AKT, p38-MAPk and JNK-SAPK from astrocytes. Vpr activates these pathways by the translocation of transcription factors for the expression of inflammatory markers. Pathway 1 also indicates the Vpr induction of IL-1β, IL-8 and TNF-α via the p38 and JNK-SAPK pathway from monocyte-derived macrophages (MDMs). **B**: Pathway 2 of Vpr-induced neuroinflammation. Pathway 2 indicates the Vpr induction of TLR4, TNF-α and NF-κB via the activity of the sulfonylurea receptor 1 –transient receptor potential melastatin 4 (Sur1-Trpm4) channels in astrocytes. The increased expression of inflammatory markers is concurrent with the expression of Sur1-Trpm4, however, the exact mechanism to how it contributes to neuroinflammation has not yet been established and indicated as “?”

Thirdly, although HIV-1 interacts with several signalling molecules, the Mitogen-activated protein kinase (MAPK) pathway is of particular interest, as these kinases play a role in various cellular activities such as activation, proliferation, differentiation, survival and cytokine production [45]. In addition, the MAP kinases including p38 and c-Jun N-terminal kinase (JNK) have been shown to be involved in immunoregulation and controlling the production and secretion of various cytokines [46]. The findings presented in this review indicate that Vpr induces inflammation trough specific pathways and channels (Fig. 3A-Fig. 3B). Specifically, the presence of HIV-1 (Vpr-positive wild type) resulted in higher production of the immune markers IL-1β, IL-8 and TNF-α compared to HIV-1 (Vpr deleted mutant), and this was attributed to increased activation of p38 and stress-activated protein kinase (SAPK)/JNK in HIV-1 (Vpr-positive wild type)-infected MDMs [36] (Fig. 3A). These elevated levels of immune markers were observed in the presence of other viral proteins, including gp120 and Tat, providing evidence for an indirect role of Vpr in inducing neuroinflammation [36]. The involvement of Vpr-induced inflammation in these pathways was further supported by work done by Gangwani and colleagues (2013, 2015), who demonstrated that Vpr activates transcription factors NF-κB, activator protein (AP)-1 and CCAAT/enhancer-binding protein (C/EBP)-δ via upstream protein kinases phosphoinositide 3-kinases (PI3K)/protein kinase B (Akt), p38-MAPK, and JNK-MAPK, leading to the induction of CCL5, IL-6 and IL-8 in astrocytes [25, 38] (Fig. 3A). Another pathway highlighted in this review involves Trpm4, a non-selective monovalent cation channel that co-associates with Sur1 to form Sur1-Trpm4. Sur1-Trpm4 channels are known to be involved in the neuropathology of several disorders [47]. A recent study reported that HIV-1 Vpr-induced pro-inflammatory responses (TLR4, TNF-α, NF-κB) and apoptosis were mediated through the Sur1-Trpm4 channel in astrocytes [40] (Fig. 3B).

Fourthly, specific amino acids in the Vpr protein have been linked to higher levels of neuroinflammation and/or neuronal apoptosis. Na and colleagues (2011) investigated post-mortem brain tissue as a preliminary investigation before proceeding to fundamental approaches. The authors found that specific signatures, Vpr 77Q, were highly prevalent in human brains with dementia (17/18), whereas Vpr 77R was more prevalent in non-demented brains (7/9) [39]. Therefore, they aimed to map and investigate the mechanisms of these specific mutations (full-length Vpr and Vpr-mutated peptides, Vpr 77Q and Vpr 77R) using in vitro and in vivo models. An unexpected finding was reported, indicating that Vpr 77R myeloid transfected cells induced significantly more TNF-α and IFN-α compared to Vpr 77Q, Vpr-null (non-expressing vector), and mock exposed (HIV-1 envelope-defective proviral plasmid) microglia. Furthermore, Vpr 77R showed the greatest neurotoxicity compared to Vpr 77Q and Vpr-null, in line with the neuroimmune response [39]. Supernatants derived from Vpr77R-and Vpr77Q transfected myeloid cells caused significant reductions in neuronal viability as measured by β-tubulin immunoreactivity in HFNs, compared with supernatants from the Vpr-null transfected cells. Thereafter, full-length Vpr (amino acids 1–96) and Vpr-mutated peptides (Vpr 77Q and Vpr 77R) were stereotactically implanted into the striatum of mice. Although the study did not directly measure inflammatory markers in the brain of animals, ionized calcium-binding adapter molecule 1 (Iba-1) was measured in relation to neuronal damage. Iba-1 is a marker for microglia activation, which plays a key role in mediating inflammation during HIV-1 infection [48]. Minimal Iba-1 immunoreactivity was observed in the basal ganglia of animals implanted with phosphate-buffered saline (PBS), whereas the numbers of Iba-1 immunopositive microglia were increased in animals implanted with full-length Vpr- and Vpr 77R, indicating a glial response to cellular injury [39]. Iba-1 immunoreactivity did not differ between the PBS-implanted animals and the Vpr 77Q implanted animals [39]. Interestingly, a reduced number of neurons was found in animals implanted with the full-length Vpr and Vpr 77R compared to those implanted with Vpr 77Q and the control group [39].

On day 28 post-implantation of the striatum, both full-length Vpr and Vpr 77R caused a significantly increase in ipsiversive rotations (rotary behaviour) compared with PBS-implanted animals. Increased ipsiversive rotations indicate neuropathology and are specifically linked to unilateral dopamine deprivation, as shown in rodent models of striatal function [49]. Although Ferrucci, and colleagues (2013) did not directly investigate the levels of inflammatory markers in animal brains, they did investigate glutathione levels in SK-N-SH neuroblastoma cells exposed to rVpr or mutant rVpr (R73 and A80) conditioned media. Glutathione is a controller of redox balance [50], and a redox imbalance may contribute to the levels of inflammation levels in HIV-1 infection [50]. Exposure to rVpr or mutant rVpr (R73 and A80) conditioned medium resulted in decreased synthesis of glutathione and increased apoptosis (measured by the number of apoptotic nuclei) compared to untreated SK-N-SH neuroblastoma cells [26].

The studies included in this review collectively reported that Vpr can independently contribute to dysregulated neuroinflammatory levels and can directly induce neuronal apoptosis. However, these studies did not determine whether Vpr-induced neuroinflammation is responsible for the observed neuronal damage or if the observed neuronal damage is primarily due to the direct neurotoxicity of Vpr. One study, however, reported reduced neuronal cell death when the levels of Vpr-induced neuroinflammation were lower [39]. Although this study did not demonstrate that Vpr-induced inflammation mediates the observed neuronal toxicity, it is reasonable to speculate that there may be a relationship between increased Vpr-induced neuroinflammation and neuronal damage. However, the extent to which Vpr-induced neuroinflammation contributes to neuronal damage is still unknown.

Lastly, due to the limited number and heterogeneity of existing studies that investigated Vpr-induced neuroinflammation, a full systematic review/meta-analysis could not be conducted. Despite this, this review disseminates the most relevant research findings to date, which may provide a basis for future studies in understanding the role of Vpr-induced neuroinflammation in the development of neuronal damage and HAND.

## Discussion

Here we have reviewed and synthesized ten fundamental studies investigating Vpr-induced neuroinflammation. The included studies represent the scope of available research literature on this topic, albeit limited, and suggest an increasing amount of evidence supporting the potential role of Vpr in the neuroinflammatory mechanism of HIV-1. As highlighted in the reviewed studies, the main finding was that soluble Vpr contributes to neuroinflammation, with studies consistently reporting higher levels of TNF-α and IL-8 in the presence of Vpr. Additional findings were that (1) Vpr induces inflammation via specific pathways, including the PI3K/AKT, p38-MAPk, JNK-SAPK and the Sur1-Trpm4 channel in astrocytes and the p38 and JNK-SAPK pathways in myeloid cells, and that (2) the Vpr protein amino acid signatures (73R, 77R and 80A) may play an important role in exacerbating neuroinflammation and the neuropathophysiology of HAND.

First, from the reviewed studies, IL-6, IL-1β, TNF-α, and IL-8 were the most commonly investigated markers. The levels of IL-6 and IL-1β varied, with cells exposed/transfected with Vpr producing or secreting both higher and lower levels of the respective interleukins compared to control cells. It is plausible to suggest these noted variances may be due to the investigated cell type for IL-6 (astrocytic cell lines, HFAs and monocytes) and IL-1β (astrocytes, MDMs and microglia). Additionally, it is relevant to note that studies investigating these markers also differed in the mode of exposure (HIV-1 infected cells (Vpr-positive vs. Vpr-negative) and/or extracellular Vpr exposure), duration of HIV-1/Vpr exposure, the concentration of HIV-1 (viral titre)/Vpr, and the technique/assay used to measure the markers (Supplementary Table 2), all of which may have influenced the findings in the reported studies.

In contrast, studies consistently reported higher levels of TNF-α and IL-8 in astrocytes and myeloid cells exposed or transfected with Vpr. Given that soluble Vpr circulates in the brain and the CNS of PLHIV [22, 39] and contributes to increased levels of TNF and IL-8 in the cells of the CNS [26, 38, 40], this suggests that Vpr may play a role in dysregulating neuroinflammation in PLHIV. In previous reviews conducted by our group, we reported that TNF-α (blood) and IL-8 (CSF and blood) levels were consistently higher in PLHIV and were associated with neurocognitive impairment [51, 52]. These markers may therefore be of interest for future studies aiming to study the potential role of inflammation in Vpr’s effects on neuronal damage and neurocognitive impairment in PLHIV.

Second, in MDMs, Vpr activated the p38 and JNK-SAPK pathway, resulting in increased production of IL-1β, IL-8 and TNF-α [36]. While the activation of the p38 and JNK-SAPK has been established to involve Vpr-induced inflammation, the exact pathway for this has not yet been elucidated. One plausible explanation is that activation of these signalling molecules may phosphorylate and/or translocate transcription factors that may activate the promoters of these inflammatory markers in MDMs. Several HIV-1 viral proteins, such as gp120 [5, 53], Nef [54, 55] and Tat [56, 57], are known to interact with MAPK pathways. Previous studies have shown that the induction of IL-1β, IL-8 and TNF-α gene expression has been linked to the MAPK pathway [45, 58]. Therefore, future studies should further investigate the exact MAPK pathways related to Vpr-induced neuroinflammation as anchor points in the development of therapeutics to inhibit Vpr-induced neuroinflammation and potential neuronal damage.

The signalling pathways responsible for the induction of IL-6, IL-8 and CCL5 in astrocytes include the activation of NF-κB, AP-1 and C/EBP-δ. In addition, the Vpr-induced production of TNF-α from astrocytes has been linked to increased expression of the Sur1-Trpm4 channel. However, the exact mechanism between the upregulation of the Sur1-Trpm4 channel and Vpr-induced neuroinflammation (i.e., TNF-α) has not been established. Increased expression of functional Sur1-Trpm4 channels may result in excessive sodium (Na+) influx into cells, leading to cell swelling, which in turn contributes to necrotic cell death and neuroinflammation. This was shown to be the case in many forms of CNS injury in preclinical models of cerebral ischemia, traumatic brain injury, spinal cord injury, and subarachnoid haemorrhage [44]. Further, pharmacological blocking of the Sur1-Trpm4 channel has been shown to attenuate neuroinflammation and neurodegeneration in the above-mentioned animal models [44]. As shown by Li and Colleagues (2020), the Sur1-Trpm4 inhibitor glibenclamide suppressed Vpr-induced apoptosis in a Vpr concentration-dependent manner in SNB19 cultures. However, it is not clear if this Vpr-induced apoptosis is directly related to the Vpr-induced neuroinflammation in astrocytes [40].

Third, several mutations in Vpr have been linked to mechanisms of HIV-1 pathology and potential immune escape of the virus. Here, findings reported that Vpr protein amino acid signatures (73R, 77R and 80A) may play an important role in the Vpr-induced neuroinflammatory mechanism of HIV-1. However, there is limited evidence from investigations on these specific Vpr mutations in the neuropathological mechanisms of HIV-1. One clinical study suggested that 73R had no contribution to the disease progression in PLHIV [59]. However, the role of this amino acid signature on neurocognitive outcomes remains unclear. Both 73R and 77R are the dominant amino acids of the NL4-3 Vpr, which is similar to the HIV-1 subtype B consensus Vpr sequence [60], and 77R is designated as important in viral function [60]. Furthermore, 77R was linked to increased neuroinflammation, however, it was less prevalent in brains affected by HAD [39]. This finding does not match the general consensus that that increased neuroinflammation is associated with worse neurocognitive outcomes [51]. The authors suggested several possible reasons for these findings, including that (1) the 77Q mutation somehow permits HIV-1 to replicate and persist in the brain by restricting the neuroimmune response, thereby augmenting the virus’ fitness and replicative capacity and resulting in higher detection in HAD brains, and (2) 77Q may perform some as-yet-unrecognized activity on the virus itself, which may not be crucial in neuroinflammation but crucial for other neurovirulent activity[39]. However, this warrants further investigation.

Vpr 80A is an uncommon, naturally occurring mutation, as the majority of HIV-1 subtypes present with the Vpr 80R [60, 61]. The Vpr R80A mutation is often synthetically introduced to investigate fundamental pathogenic mechanisms [61–63]. Although there is limited evidence supporting the role of these mutations in neuroinflammation and neuronal damage, this preliminary evidence provides a starting point for future investigation. This is particularly relevant given the increasing evidence that Vpr-specific residues are related to neurocognitive impairment in PLHIV. In previous clinical studies, it was noted that Vpr amino acids N41 and A55 were associated with neurocognitive impairment [12]. These amino acid signatures were also investigated by a study done by Womersley and colleagues (2019), but they found no significant effects of the amino acids N41 and A55 on global cognitive function. However, they did show that these protein signatures explained the variance in cognitive function [33]. Limited studies have investigated Vpr sequence variation in HIV-infected participants, and even fewer studies have investigated the role of Vpr amino acid variation in HIV-1 neuropathogenesis. It is therefore unclear how these Vpr signatures may contribute to neuronal damage and the various underlying mechanisms of HAND, including transactivation, inflammation, metabolism, BBB damage and neuronal damage.

Lastly, Vpr has been shown independent contribute to increased neuroinflammation, even in the presence of other viral proteins including Tat and gp120, which are well-established contributors to neuroinflammation [36]. Although the studies reviewed here were fundamental in design, we hypothesize that Vpr-induced neuroinflammatory mechanisms may be active and contribute to the dysregulated immune profile experienced in PLHIV, potentially be a contributing to the development of HAND. At this stage, the direct relationship between Vpr-induced neuroinflammation, neuronal damage and HAND has not yet been established. However, preliminary evidence from a fundamental study showed that when myeloid cells were transfected with Vpr variants, the variants that induced the highest level of neuroinflammation also had the highest level of neurotoxicity [39]. In this instance, it should be evaluated whether Vpr-induced neuroinflammation is driving neuronal damage or if the observed neuroinflammation is a result of direct Vpr-induced neuronal damage. Therefore, this traditional accessory protein deserves more emphasis for its potential role in the development of HAND.

## Recommendations

Based on the findings reported here, several recommendations could be made. First, we acknowledge that a limited number of fundamental studies are available that have investigated Vpr-induced neuroinflammation, and therefore any suggestions highlighted here were made in light of the limited investigations available. Despite this, we believe that this review provides a comprehensive overview of the literature on this topic and highlights the need for further investigation to determine the exact mechanisms of Vpr-induced neuroinflammation. This may aid in further understanding the potential contribution of Vpr to neuroinflammation and potential neuronal damage. Secondly, although studies have demonstrated that mutations in HIV-1 Vpr could dramatically affect its known functions [64–66], these mutations were artificially created and do not represent the profile of naturally occurring mutations throughout the course of infection. In support of this, a recent systematic review by our group [67] has highlighted that to date, only 22 clinical studies have investigated Vpr sequence variation and clinical outcomes in PLHIV. Therefore, there is a need to investigate the influence of Vpr sequence variation in clinical sample types (e.g., blood). In addition, Vpr-specific mutations across subtypes have not been clearly defined, and the influence of these subtype-specific Vpr mutations on mechanisms related to HIV-1 neuropathogenesis requires further investigation. Lastly, studies that investigated the relationship between Vpr presence, neuroinflammation and neurocognitive performance in PLHIV are lacking. Therefore, it is unclear whether Vpr-induced inflammation may contribute to clinical neurocognitive impairment in PLHIV. This is an area that should be addressed through studies using multimodal approaches that include neuroimaging, neuroinflammation and neurocognitive evaluation.

## Conclusions

Here we report from the available evidence that the HIV-1 viral protein Vpr may be an important contributor to neuroinflammation. Specifically, the presence and activity of Vpr have been linked to higher levels of TNF-α and IL-8. Vpr induces neuroinflammation via specific pathways, including the PI3K/AKT, p38-MAPk, JNK-SAPK and Sur1-Trpm4 channels in astrocytes and the p38 and JNK-SAPK in myeloid cells. Specific Vpr amino acids, including 73R, 77R and 80A, may be important in modulating neuroinflammation and should be investigated in future studies. The Vpr-induced markers, pathways, and amino acid signatures highlighted in this review should be investigated for their potential roles in the neuropathogenesis of HAND.

### Electronic supplementary material

Below is the link to the electronic supplementary material.

Supplementary Material 1

Supplementary Material 2

Supplementary Material 3

## Data Availability

Data sharing not applicable to this article as no datasets were generated or analysed during the current study. All supporting data (Supplementary files/tables) is attached to this manuscript.
