# Supplementary material for: Viral protein R (Vpr)-induced neuroinflammation and its potential contribution to neuronal dysfunction: a scoping review
Source: BMC Infect Dis. 2023 Aug 6;23:512. doi: 10.1186/s12879-023-08495-3 (PMC10405499; doi:10.1186/s12879-023-08495-3)
Supplement: Supplementary file 1 — Supplementary Material 1 [file 12879_2023_8495_MOESM1_ESM.docx]

**Supplementary table 1**: Quality control of included studies

| **Reference** | **Question 1** | **Question 2** | **Question 3** | **Question 4** | **Rating** |
| --- | --- | --- | --- | --- | --- |
| ^[23]^ | 2 | 2 | 2 | 2 | High |
| ^[26]^ | 2 | 2 | 2 | 2 | High |
| ^[38]^ | 2 | 2 | 2 | 2 | High |
| ^[25]^ | 2 | 2 | 2 | 2 | High |
| ^[36]^ | 2 | 2 | 2 | 2 | High |
| ^[22]^ | 2 | 2 | 2 | 1 | High |
| ^[40]^ | 2 | 2 | 2 | 2 | High |
| [32] | 2 | 2 | 2 | 1 | High |
| ^[39]^ | 2 | 2 | 2 | 2 | High |
| ^[37]^ | 2 | 2 | 1 | 2 | High |

Questions 1-4 were classified as follows: 1) Is it clear in the study what is the ‘cause’ and what is the ‘effect’ (i.e., there is no confusion about which variable comes first)? 2) Was there a control group? 3) Were there multiple measurements of the outcome both pre and post the intervention/exposure? And 4) Were outcomes measured in a reliable way? Studies with ratings between 6 and 8 were classified as high quality. Studies with ratings between 3 and 5 were considered as intermediate quality, and between 0 and 2 as low quality
