# Supplementary material for: Viral protein R (Vpr)-induced neuroinflammation and its potential contribution to neuronal dysfunction: a scoping review
Source: BMC Infect Dis. 2023 Aug 6;23:512. doi: 10.1186/s12879-023-08495-3 (PMC10405499; doi:10.1186/s12879-023-08495-3)
Supplement: Supplementary file 2 — Supplementary Material 2 [file 12879_2023_8495_MOESM2_ESM.docx]

**Pubmed: 164 (27/02/2023):**

(Viral protein R [tw] OR Gene Products, vpr [mh]) AND (HIV associated neurocognitive disorders [mh] OR HAND [tw] OR neurocognitive [tw] OR cogniti* [tw] OR Neuropsychological Tests [mh] OR neuronal damage [tw] OR neuronal apoptosis [tw] OR inflammation [mh] OR Cytokines [mh] OR Chemokines [mh] OR Neurogenic Inflammation [mh] OR neuroinflammation [tw] OR TNF [tw] OR Interleukins [mh] OR interleukins [tw] OR Microglia [mh] OR Monocytes [mh] OR Microglia [mh] OR microglia [tw] OR Monocytes [mh] OR monocyte* [tw] OR sCD163 [tw] OR sCD14 [tw] OR sCD40 [tw] OR Neopterin [mh] OR Interferons [mh])

**Web of science: 92 (27/02/2023):**

TS=(Viral Protein R OR VPR) AND TS=(HIV associated neurocognitive disorders OR HAND OR neurocognitive OR cogniti* OR Executive Function OR executive OR Memory OR memory OR Attention OR attention OR Neuropsychological Tests OR neuronal damage OR neuronal apoptosis) AND TS=(Cytokines OR cytokin* OR Chemokines OR chemokine OR Inflammation OR inflammation OR Neurogenic Inflammation OR neuroinflammation OR TNF OR Interleukins OR interleukins OR Microglia OR microglia OR Monocytes OR monocyte* OR sCD163 OR sCD14 OR sCD40 OR neopterin OR interferons)

**Scopus: 1379 (27/02/2023):**

(Viral protein R OR vpr ) AND ( hiv associated neurocognitive disorders OR hand OR neurocognitive OR cogniti* OR executive AND function OR executive OR memory OR memory OR attention OR attention OR neuropsychological AND tests OR neuronal AND damage OR neuronal AND apoptosis ) AND ( cytokines OR cytokin* OR chemokines OR chemokine OR inflammation OR neurogenic AND inflammation OR neuroinflammation OR tnf OR interleukins OR interleukins OR microglia OR microglia OR monocytes OR monocyte* OR scd163 OR scd14 OR scd40 OR neopterin OR interferons)

**Total: 1635**
