# Supplementary material for: Viral protein R (Vpr)-induced neuroinflammation and its potential contribution to neuronal dysfunction: a scoping review
Source: BMC Infect Dis. 2023 Aug 6;23:512. doi: 10.1186/s12879-023-08495-3 (PMC10405499; doi:10.1186/s12879-023-08495-3)
Supplement: Supplementary file 3 — Supplementary Material 3 [file 12879_2023_8495_MOESM3_ESM.docx]

**Supplementary Table 2**: Summary table reporting potential factors that may have influenced findings in the reported studies.

| **Cell type** | **Mode of exposure** | **Time of Vpr exposure investigated** | | | | **Concentration-dependent effects investigated** | | | | **Cell differentiation reported** | | | **Technique/assay** | **Reference** |
| --- | --- | --- | --- | --- | --- | --- | --- | --- | --- | --- | --- | --- | --- | --- |
|  |  | **Yes** | **No** | **Duration** | **Influence** | **Yes** | **No** | **Concentration** | **Influence** | **Yes** | **No** | **Duration** |  |  |
| Microglia | HIV-1 infected microglia (Viral protein R (Vpr)+ and Vpr-) | X |  | 5, 12 and 19 days | C-C chemokine ligand (CCL)5 was significantly higher at all time points compared to Vpr- infected microglia |  | X | 40ng of HIV-infected cells as measured p24 | N/A |  | X |  | Enzyme-linked immunosorbent assay (ELISA) | [37] |
| Monocyte-derived macrophages (MDMs) | HIV-1 infected MDMs (Vpr+ and Vpr-) | X |  | 1, 2, 3, 4, 8, 12, 16, 20 days | Interleukin (IL)-8 and IL-1β were the only markers elevated at all time points compared to Vpr- infected microglia |  | X | N/A | N/A | X |  | 7–8-day old MDMs | ELISA | [36] |
| Macrophages | HIV-1 infected microglia (Vpr+ and Vpr-) and Vpr exposure |  | X | 24 hours | N/A | X |  | 0, 25, 50, 100, 200, 400 ng of Vpr | IL-1β peaked at 100ng and decreased slightly at 200-400ng of Vpr |  | x |  | Immunofluorescence | [32] |
| Myeloid | Transfection |  | X |  | N/A |  | X |  | N/A |  | X |  | Polymerase chain reaction (PCR) | [39] |
| Microvascular endothelial cells (MVECs) | Exposure to extracellular Vpr |  | X | 48 hours | N/A |  | X | 50ng/ml of Vpr | N/A |  | X |  | ELISA | [23] |
| Monocytes and astrocytes | Exposure to extracellular Vpr |  | X | 6 hours | N/A | X |  | 0.1, 1, 10 and 100nM | IL-1β levels from astrocytes decreased with increased Vpr compared to controls. IL-6 was significantly higher than controls only at 100nM Vpr. IL-1β concentrations from monocytes were significantly higher at 0.1 and 10nM Vpr. IL-6 was not significantly higher different compared to controls at any concentration of Vpr. | X |  | Astrocytes: 3 days | PCR | [22] |
| Human fetal astrocytes (HFAs) | Exposure to extracellular Vpr |  | X | 48 hours | N/A |  | X | N/A | N/A |  | X | N/A | Cytokine Array | [26] |
| HFAs | Transfection | X |  | 1,  3, 6, 12, 24, 48 and 72 hours | The CCL5 mRNA expression level peaked  at 3 (24.43 ± 3.18-fold) and declined thereafter to reach  the basal level at 48 h. The CCL5 protein concentration was  higher at all time intervals analysed, and the peak CCL5  concentration was seen at 48 h post-transfection (2,040.5 ±  209.6 pg/ml) compared to mock-transfected controls  (200.7 ± 20.1 pg/ml). |  | X | N/A | N/A |  | X | N/A | Polymerase chain reaction (PCR) and Immunocytochemistry | [25] |
| HFAs | Transfection | X |  | 1,  3, 6, 12, 24, 48 and 72 hours | The peak IL-6 and IL-8 messenger (m)RNA was found at 12h (20.03 ± 4.13-Fold) and 6h  (16.36 ± 1.86-Fold) post-transfection as compared to mock-transfected controls. The secreted IL-6 and IL-8 protein levels were increased at all-time points assayed with a peak  at 72h (10,762 ± 3230 pg/ml; 1287 ± 178 pg/ml), as compared to mock-transfected controls  (2495 ± 1826 pg/ml; 385 ± 34 pg/ml), respectively. |  | X | N/A | N/A |  | X | N/A | Polymerase chain reaction (PCR) | [38] |
| Astrocytes | Transfection |  | X |  | N/A | X |  |  | Vpr increased, in an MOI-dependent manner, the transcription of genes encoding TLR4, TNF-  a, and the Sur1-encoding Abcc8 gene.  Transcriptional expression of the gene encoding NF-κB was also increased with the  increase of Vpr concentration; however, due to high experimental variability, the MOI-response relationship did not reach statistical significance. |  | X | N/A | Immunohistochemistry | [40] |

Abbreviations: C-C chemokine ligand (CCL), Enzyme-linked immunosorbent assay (ELISA), Interleukin (IL), Messenger RNA (mRNA), Monocyte-derived macrophages (MDMs), Polymerase chain reaction (PCR) and Viral protein R (Vpr)
